# A Cost-Efficient Method for Unsymmetrical *Meso*-Aryl Porphyrin Synthesis Using NaY Zeolite as an Inorganic Acid Catalyst

**DOI:** 10.3390/molecules22050741

**Published:** 2017-05-05

**Authors:** Mário J. F. Calvete, Lucas D. Dias, César A. Henriques, Sara M. A. Pinto, Rui M. B. Carrilho, Mariette M. Pereira

**Affiliations:** Coimbra Chemistry Centre, CQC, Departamento de Química, Faculdade de Ciências e Tecnologia da Universidade de Coimbra, Rua Larga, 3004-535 Coimbra, Portugal; lucasdanillodias@gmail.com (L.D.D.); c.henriques@qui.uc.pt (C.A.H.); smpinto@qui.uc.pt (S.M.A.P.); rui.carrilho@uc.pt (R.M.B.C.)

**Keywords:** unsymmetrical porphyrins, inorganic acid catalysis, alternative synthetic methodologies

## Abstract

Herein we report the synthesis of unsymmetrical *meso*-aryl substituted porphyrins, using NaY zeolite as an inorganic acid catalyst. A comparative study between this method and the several synthetic strategies available in the literature was carried out. Our method presented a better, more cost-efficient rationale and displayed a significantly lower environmental impact. Furthermore, it was possible to verify the scalability of the process as well as the reutilization of the inorganic catalyst NaY (up to 6 times) without significant yield decrease. In addition, this method was applied to the synthesis of several other unsymmetrical porphyrins, from a low melting point porphyrin to mono-carboxylated halogenated unsymmetrical porphyrins, in yields higher than those found in the literature. Additionally, for the first time, two acetamide functionalized halogenated porphyrins were prepared in high yields. This methodology opens the way to the preparation of high yielding functionalized porphyrins, which can be easily immobilized for a variety of applications, either in catalysis or in biomedicine.

## 1. Introduction

Owing to porphyrin’s myriad of applications, including biomedicine [1,2,3,4,5,6,7,8], catalysis [9,10,11,12], and materials [13,14,15,16,17,18], the preparation of unsymmetrically substituted porphyrins, bearing bridgeable groups for further linkage to other chemical entities and materials, is of utmost interest [19,20,21,22,23,24,25,26]. Unsymmetrically substituted porphyrin design has been applied for several decades, at first, using porphyrin β-pyrrolic substituting patterns such as the 3+1 route [27,28,29], employing the chemistry proposed in the well-known MacDonald 2+2 method [30], which essentially relied on the cumbersome synthesis of tripyrranes [31], later mitigated by Sessler’s advances on their syntheses [32]. Despite all progress [33,34], over the last decade, porphyrin scientists have turned their attention to the preparation of *meso*-patterned unsymmetrical porphyrins, mostly given to their more amenable syntheses [35,36,37,38,39], but still presenting a high number of steps in their methodologies (low overall yields), despite their lower need of purification procedures. Nevertheless, classical methods are the most used for synthesizing the commonly denominated A_3_B-*meso*-patterned porphyrins, which rely on mixed aldehyde condensation in a 3:1 proportion (hence A_3_B) with four equivalents of pyrrole. Under the specific conditions of each method, this approach produces a statistical mixture of products that have to be further separated from the desired compound; enough reason to assume the imperative necessity to develop cost-efficient methods for the preparation of unsymmetrically substituted porphyrins on a larger scale.

Hence, the development of profitable synthetic methodologies for the preparation of porphyrins bearing at least one bridgeable chemical group, concomitantly with other property-enhancing groups, is presumably one of the main aims in synthetic porphyrin chemistry at present.

Herein, we expand the scope and demonstrate the versatility of our recent synthetic method, which uses a porous solid inorganic acid (zeolite NaY) as catalyst [40,41,42] to promote the cyclization of pyrrole with a series of aromatic aldehydes, bearing electron donating or withdrawing substituents, portraying a highly improved yield synthesis of several unsymmetrical *meso*-aryl substituted porphyrins, some of them for the first time. We also discuss the features of this new methodology for the preparation of unsymmetrical *meso*-aryl porphyrins from an ecological and economical point of view, which are crucial aspects when choosing a methodology for the preparation of such key synthons, with a multitude of applications.

## 2. Results and Discussion

### 2.1. Synthesis and Characterization of Unsymmetrically Substituted Porphyrins

It is well established that the pKa and the type of acid (organic [43,44,45] or solid [46,47]—Lewis or Brønsted) are determining factors for efficient pyrrole and aldehyde condensation. Using acids as catalysts, the current methods for *meso*-aryl substituted porphyrin syntheses include the classical one-pot Adler-Longo [43] and Gonsalves-Pereira [44] strategies and the two-step Lindsey method [45], along with more recent microwave irradiation methodologies [48,49,50,51,52].

Following our previously reported method, where NaY was used as a porous solid acid catalyst [40], and since the legitimate establishment of any porphyrin synthetic methodology implies the demonstration of its flexibility and general usability, we established first a direct correlation between applied methodologies and the yields of desired compounds.

Therefore, we carried out the preparation of 5-(4-hydroxyphenyl)-10,15,20-tris(phenyl) porphyrin (**1**) [8,17,23,53] as the model unsymmetrical *meso*-aryl porphyrin using the three best established methods against our herein disclosed methodology (Table 1). For the sake of comparison, we scrupulously followed the literature-described reaction conditions of Adler-Longo [43], Gonsalves-Pereira [44], and Lindsey [45] methods (see Supplementary Materials—Section S3).

**Scheme 1:**
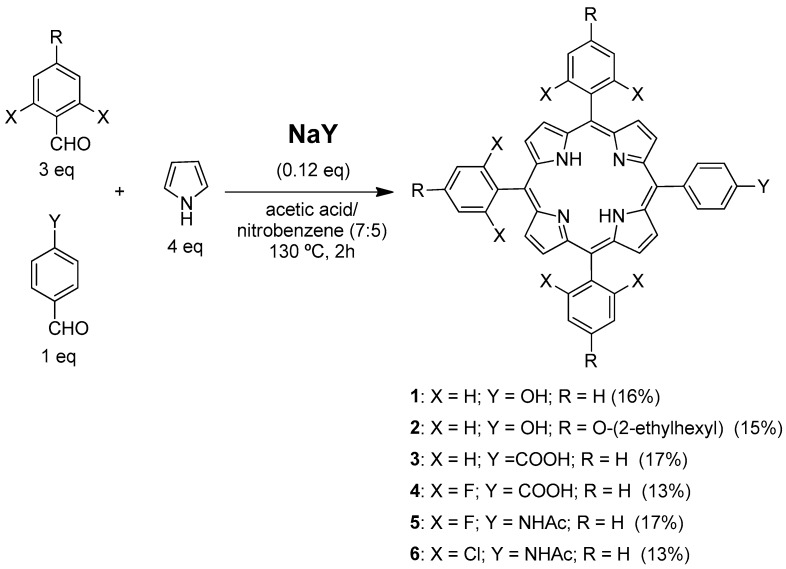
Synthesis of unsymmetrical *meso*-aryl substituted porphyrins, using NaY zeolite as an inorganic acid catalyst.

For our “NaY” method [40], in a typical experiment, 0.016 molar equivalents of NaY were added to 3 molar equivalents of benzaldehyde, 1 molar equivalent of 4-hydroxybenzaldehyde, and 4 molar equivalents of pyrrole mixed in glacial acetic acid and nitrobenzene (7:5) in a 0.42 molar concentration. The reaction mixture was heated at 130 °C for ca. 2 h (Scheme 1). After an experimental workup, which included column chromatography using silica as a stationary phase, the title compound **1** was isolated in 16% yield (see Material and Methods and Supplementary Materials—Section S3 for details).

**Figure 1:**
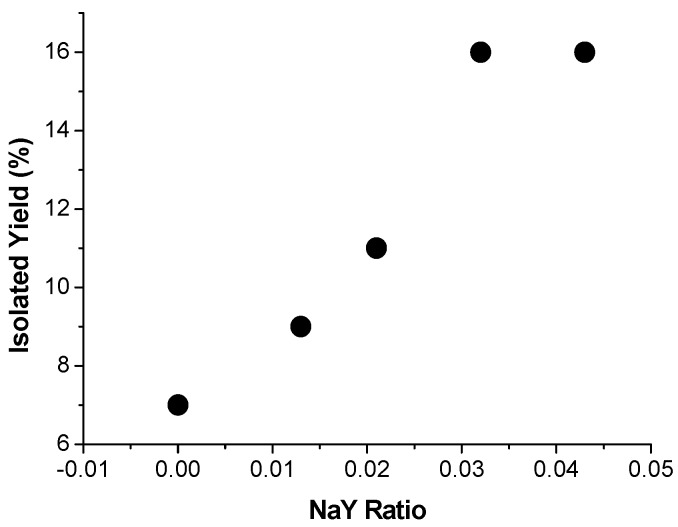
Isolated yields of Porphyrin **1** vs. the NaY amount. Note: when NaY ratio = 0, it represents the isolated yields using the Gonsalves-Pereira method.

Analysis of Table 1 shows that using the Adler-Longo or Gonsalves-Pereira methods, the yields for obtaining porphyrin **1** are quite similar (6 and 7% yields, respectively). The main difference between these two strategies resides in that, using the Adler-Longo method, the yield is overestimated by the contamination with the corresponding chlorin (around 10–15%, by UV-Vis checking), which could be further oxidized to its correpsonding porphyrin, but was out of the scope in this study. On the other hand, the Gonsalves-Pereira method permitted the isolation of porphyrin **1** with high purity, without chlorin contamination, in a slightly higher yield. Lindsey methodology provided the desired compound in 15% yield, but using quite diluted reaction conditions (0.03 M) and the expensive DDQ oxidant, involving two steps, while our methodology, which uses NaY as co-catalyst, provided the same compound in 16% yield, using concentrations of 0.42 M and a simpler procedure. In our case, the reaction of pyrrole and aldehydes, using a mixture of acetic acid and nitrobenzene as a solvent (acetic acid works also as catalyst and nitrobenzene as the oxidant), in the presence of porous solid NaY zeolite in ca. 3.2 mol %, as co-catalyst, enhanced yield of the desired unsymmetrical *meso*-aryl substituted porphyrin **1**. Lewis acidity values were calculated for NaY by the pyridine (Py) adsorption method, followed by infrared spectroscopy, giving a value of 520 mmol Py per g of NaY (see Supplementary Materials—Section S2), which attests its high capacity to act as a Lewis acid in the reaction condensation between pyrrole and aldehydes. It is worth mentioning that the ratio between NaY and all reagents (pyrrole and aldehydes (3.2 mol %) (assuming NaY MW = 12,752 g/mol) [54] was established after tests using different ratios (Figure 1 and Supplementary Materials—Section S2).

When a 2.1 mol % NaY was used, the yield of porphyrin **1** was 11%, while a yield of 16% was obtained when the ratio was increased to 4.3 mol %, identical to the yield obtained, when a 3.2 mol % amount of NaY was used. Therefore, it was demonstrated that the condensation reaction in the presence of porous solid NaY zeolite as a co-catalyst at ca. 3.2 mol % held the best proportion from a cost-efficiency point of view. Moreover, it should be mentioned that this synthesis was easily scalable from 2.5 to 100 mmol (based on pyrrole), without any appreciable drop on the overall yield, demonstrating the high scalability of the method (see Supplementary Materials—Section S4).

**Figure 2:**
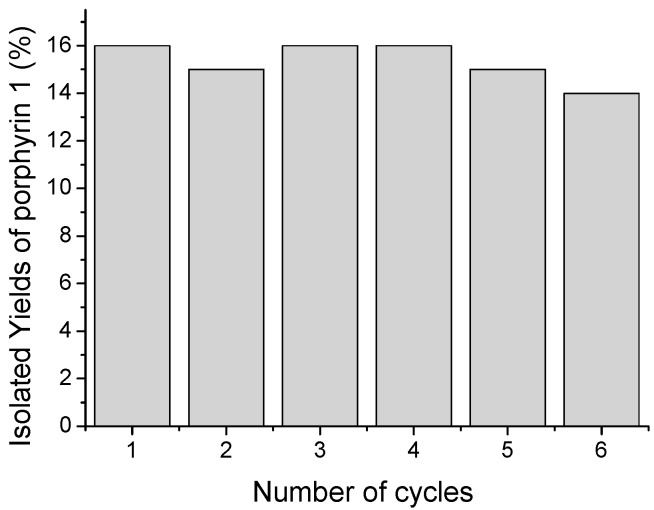
Reutilization of NaY on the synthesis of Porphyrin **1**.

However, the application of this method as a real practical alternative for the synthesis of unsymmetrical porphyrins requires the validation of NaY as a reusable catalyst. This was accomplished by evaluating its reutilization in the synthesis of Porphyrin **1** (Figure 2) (see Supplementary Materials—Section S5).

After the first cycle, the NaY catalyst was collected by filtration and washed with dichloromethane and tetrahydrofuran, followed by drying of the solid overnight, at 150 °C under vacuum. The subsequent cycles were carried out with the recovered solid and reactivation procedure was similar for each reutilization. We also carried out a reutilization experiment where the catalyst was used without prior reactivation, yielding Porphyrin **1** in a 9% yield, similar to that obtained using the Gonsalves-Pereira method alone (7%). This observation suggests that NaY voids are mainly occupied by organic materials, which may block the catalyst’s acid sites.

### 2.2. The Determination of Method’s Sustainability and Cost-Efficiency

Reaction efficiency is a very important issue, but ecological impact also plays a key role when designing more sustainable synthetic methodologies. Therefore, metrics that have been developed to judge synthetic efficiency must be used [55]. An important parameter is the environment factor (E Factor), which represents the ratio between the weight of waste generated and the total weight of the end product [56,57]. We analyzed several established methodologies for the preparation of 5-(4-hydroxyphenyl)-10,15,20-tris(phenyl) porphyrin (**1**), obtaining approximate E Factor values for each method (see Table 2 and Supplementary Materials—Section S6). The calculation of E Factors was carried out considering the production of 10 mmol of Porphyrin **1**, i.e., 6.3 g. To obtain this amount of end product, we correlated the yield obtained for each reaction with each solvent/reagent used.

Looking at E Factors, it is clear that the two-step Lindsey methodology is a higher waste-producer, expectable from the inherent high dilution requirements (E Factor = 7090), while reactions using a high reagent concentration hold a lower E Factor (Table 2). It must be noted that, in a report by Lindsey and co-workers [58], where high concentration conditions were employed, an E factor of 2750 was calculated (see Supplementary Materials), demonstrating the importance of concentration in the waste production of such type of reactions. Considering the production of waste, our NaY method exhibits an E Factor of 2350, a lower value than those presented by the other methods, validating the reduction of solvents used and causing an improvement in the relative eco-friendliness of the method regarding waste production. However, it should be noted that a very significant cost of waste disposal is the cost of the disposal of silica, which is used in considerable amounts in statistical methodologies. Considering the approximations taken for the E Factor, we calculated, at current market prices, the cost of the production of 10 mmol of Porphyrin **1** (Table 2), in order to account for the cost-efficiency of our methodology. We point out that the cost of labor, which is often a determining factor in choosing a scale-up procedure, is not evaluated here since it is similar for all statistical procedures and would similarly affect all methods. When designing a potential scale-up process, one must consider, when possible, the reutilization of solvents, particularly when used as pure solvents, not mixtures. Considering the reutilization of reaction and chromatography solvents, along with catalysts, the comparison of costs among the various methods changed considerably, as Lindsey’s method prices reduced to less than half, for instance. Price analysis proves that the “NaY method,” when compared to the other methodologies, is the most suitable method for the production of assessable quantities of Porphyrin **1**, with a substantially favorable cost-to-efficiency ratio of 504 € per 10 mmol porphyrin (6.3 g), i.e., 80 € per gram (see Supplementary Materials—Section S7).

### 2.3. Scope

Having in hand a methodology displaying a cost-efficient feature, we hypothesized that extrapolation to the preparation of other unsymmetrical *meso*-aryl substituted porphyrins could be achieved. Thus, we extended the application of the NaY method to the preparation of Porphyrins **2**–**6** (Scheme 1) (see Supplementary Materials—Section S8).

Considering our interest in highly soluble low melting point porphyrins [41,52], we prepared 5-(4-hydroxyphenyl)-10,15,20-tris(4-ethylhexyloxyphenyl) porphyrin (**2**) in 15% yield, higher than the previously reported using other methodologies (7–10%) [41]. Similarly, and considering the high importance of monocarboxylic acid-substituted porphyrins for further functionalization, 5-(4-carboxyphenyl)-10,15,20-tris(phenyl) porphyrin (**3**) was also prepared in 17% yield in just one step, while a careful literature search revealed that the best yields are usually obtained by first preparing the 5-(4-methoxycarbonylphenyl)-10,15,20-tris(phenyl) porphyrin, followed by ester hydrolysis, attaining 6.5% overall yields [59]. Additionally, the 5-(4-carboxyphenyl)-10,15,20-tris(2,6-difluorophenyl) porphyrin (**4**) was prepared in 13% yield, in a single step in a yield comparable to the literature (16%) [60], albeit obtained in a much higher amount when compared with the highly diluted Lindsey two-step procedure used, plus the deprotection of the corresponding methyl ester. Therefore, our method was revealed to be cost-efficient for the preparation of the above *meso*-monocarboxylphenyl substituted porphyrins using a one-step procedure, in yields comparable to those reported in the literature with obvious economical saving and environmental gain.

Finally, our herein described methodology afforded the new unsymmetrical *meso*-substituted mono-acetamide porphyrins **5** and **6**. Following our particular interest on the synthesis of bioconjugated ^19^F-NMR probes for biomedicinal applications, [61,62,63], for the first time, 5-(4-acetylaminophenyl)-10,15,20-tris(2,6-difluorophenyl) porphyrin (**5**) was prepared, in 16% yield. Likewise, aiming at the preparation of covalently immobilized catalysts, and bearing in mind the outstanding properties of halogenated porphyrins as oxidation catalysts [9,64,65], our synthetic methodology was also applied to prepare, for the first time, 5-(4-acetylaminophenyl)-10,15,20-tris(2,6-dichlorophenyl) porphyrin (**6**) in 13% yield. These results are promising considering the low yields usually obtained in the synthesis of sterically hindered porphyrins through the condensation of 2,3-disubstituted benzaldehydes and pyrrole.

## 3. Materials and Methods

Commercially available reagents were purchased from Sigma-Aldrich (Lisbon, Portugal), Fluorochem (Derbyshire, UK) and Acros (Santo Antão do Tojal, Portugal), being used as received. Zeolite NaY was purchased from Zeolyst (Groningen, The Netherlands). All solvents were pre-dried according to standard laboratory techniques. All spectroscopic data from known porphyrins were confirmed (^1^H-NMR and UV-Vis) and agree with the literature. UV-visible absorption spectra were recorded on a Hitachi U-2010 (Hitachi Corporation, Tokyo, Japan) using quartz cells. The molar absorption coefficients were determined using toluene as a solvent. Zeolite acidity measurements were performed using pyridine as probe molecule, followed by infrared spectroscopy (FTIR); a Nicolet Nexus spectrometer (GMI Inc., Ramsey, MN, USA) was used for the purpose. ^1^H-NMR spectra were recorded on a 400 MHz Brucker-Amx (Bruker, Karlsruhe, Germany). The chemical shifts are given in parts per million (ppm) relative to tetramethylsilane at δ 0.00 ppm for proton spectra. Mass spectra were acquired using an Applied Biosystems Voyager DE-STR instrument (Applied Biosystems, Foster City, CA, USA) equipped with a nitrogen laser (λ = 337 nm) or Bruker microTOFQ instrument (Bruker, Madrid, Spain) by Unidade de Espectrometría de Masas e Proteómica, Universidade de Santiago de Compostela, Spain. Column chromatographies were performed with silica gel grade 60, 70–230 mesh as a stationary phase.

Details of the synthetic procedures employed in the synthesis of Porphyrins **1**–**6** are described in Supplementary Materials. The synthetic procedure for a typical experimental using the ”NaY method” is described below.

### 3.1. General Procedure

One gram of NaY zeolite (0.08 mmol) was placed into a 50 mL round flask, containing a 1:3 mixture of aldehydes (0.625:1.875 mmol), in a glacial acetic acid/nitrobenzene mixture (7/5 mL). An addition of equimolar amount of pyrrole (2.5 mmol, 0.17 mL) was carried out dropwise under stirring and heating (≈120 °C). After the complete addition (ca. 3 min), the suspension was heated further to reflux temperature (≈130 °C) and maintained at this temperature for ca. 2 h. The hot suspension was filtered through a sintered glass filter (a porosity of 4), and the resulting solid material was washed with tetrahydrofuran (THF) until no colored material was collected on the supernatant (250 mL). The volume of the solution was then reduced by rotoevaporation (enough volume to remove the added washing solvents). To induce precipitation, *n*-hexane (ca. 50 mL) was added. The Erlenmeyer flask containing the statistical porphyrin mixture was left overnight in the refrigerator, at 4 °C, and the deposited solid was collected by filtration and then purified by column chromatography using silica gel as a stationary phase, starting with *n*-hexane/CH_2_Cl_2_ (1:3) then increasing polarity using appropriate gradients of *n*-hexane/CH_2_Cl_2_, then pure CH_2_Cl_2_, and finally CH_2_Cl_2_/ethanol gradients if necessary (details in Supplementary Materials) to collect the second fraction (the target compound, in all cases). This fraction was then evaporated to dryness, and the resulting solid was dried under vacuum and weighed. Characterization data from Compounds **1** [53], **2** [52], **3** [59], and **4** [60] are in accordance with those in the literature. The characterization data for the new compounds are displayed below.

#### 3.1.1. 5-(4-Acetylaminophenyl)-10,15,20-tris(2,6-difluorophenyl) Porphyrin (**5**)

According to the general procedure, the benzaldehydes used were as follows: 4-acetylaminobenzaldehyde (0.625 mmol, 102 mg) and 2,6-difluorobenzaldehyde (1.875 mmol, 267 mg). Column chromatography was carried out using as eluent n-hexane/CH_2_Cl_2_ (1:3) to collect the first fraction (A_4_ product) followed by ethanol/CH_2_Cl_2_ (1:100) to collect Porphyrin **5**. We obtained 78 mg of Porphyrin **5** (16% yield).

^1^H-NMR (CDCl_3_, 400 MHz), δ H (ppm) = 8.90–8.83 (m, 8H, β-H), 8.17 (d, *J* = 8.0 Hz, 2H, Ph(Ac)-H), 7.90 (d, *J* = 8.0 Hz, 2H, Ph(Ac)-H), 7.89–7.76 (m, 3H, Ph-H), 7.49–7.36 (m, 6H, Ph-H), 2.36 (s, 3H, CH_3_), −2.75 (s, 2H, NH). ^13^C-NMR (101 MHz, CDCl_3_) δ C (ppm) = 134.9, 130.8, 129.1, 119.7, 118.0, 111.5, 111.2, 24.6. ^19^F-NMR (376.5 MHz, CDCl_3_) δ F (ppm) = −108.2, −108.3.

HRMS (ESI-FIA-TOF): Calcd. for C_46_H_28_F_6_N_5_O: 780.2193; Found *m/z* = 780.2202 [M]^+^.

UV-vis (toluene): λ_max_, nm (ε, M^−1^∙cm^−1^) 420 (3.5 × 10^5^), 515 (2.3 × 10^4^), 546 (6.2 × 10^3^), 591 (6.9 × 10^3^), 656 (4.3 × 10^3^).

EA: Anal. Calcd for C_46_H_28_F_6_N_5_O: C: 70.86; H: 3.49; N: 8.98. Found: C: 70.87; H: 3.49; N: 8.97.

#### 3.1.2. 5-(4-Acetylaminophenyl)-10,15,20-tris(2,6-dichlorophenyl) Porphyrin (**6**)

According to the general procedure, the benzaldehydes used were as follows: 4-acetylaminobenzaldehyde (0.625 mmol, 102 mg) and 2,6-dichlorobenzaldehyde (1.875 mmol, 328 mg). Column chromatography was carried out using as eluent *n*-hexane/CH_2_Cl_2_ (1:3) to collect the first fraction (A_4_ product) followed by ethanol/CH_2_Cl_2_ (1:100) to collect Porphyrin **6**. We obtained 71 mg of Porphyrin **6** (13% yield).

^1^H-NMR (400 MHz, CDCl_3_), δ H(ppm) = 8.85 (d, *J* = 4.5 Hz, 2H, β-H), 8.66–8.62 (m, 6H, β-H), 8.12 (d, *J* = 8.0 Hz, 2H, Ph(Ac)-H), 7.85 (d, *J* = 7.9 Hz, 2H, Ph(Ac)-H), 7.79–7.65 (m, 9H, Ph-H), 2.31 (s, 3H, CH_3_), −2.56 (s, 2H, NH). ^13^C-NMR (101 MHz, CDCl_3_) δ C(ppm) = 135.1, 131.2, 129.2, 124.6, 119.7, 117.6, 111.6, 111.4, 24.8.

MS (MALDI-TOF): *m/z* = 879.039 [M]^+^.

UV-vis (toluene): λ_max_, nm (ε, M^−1^∙cm^−1^) 420 (3.6 × 10^5^), 514 (2.3 × 10^4^), 545 (6.1 × 10^3^), 591 (6.9 × 10^3^), 656 (4.4 × 10^3^).

EA: Anal. Calcd. for C_46_H_27_F_6_N_5_O: C: 62.89; H: 3.10; N: 7.97. Found: C: 62.85; H: 3.08; N: 7.99.

## 4. Conclusions

To conclude, the tested NaY zeolite acts as an efficient co-catalyst in the synthesis of unsymmetrical porphyrins in solution. The NaY (Lewis acidity of 520 mmol pyridine per g) also revealed high potential for the multi-gram scale preparation of unsymmetrical porphyrins, in similar or even higher yields than those reported, advantageously presenting a better, more cost-efficient rationale, along with a lower environmental impact, when compared to established methods, judging by its E Factor, a very important metric when designing environmentally sustainable methodologies. The performed approximate price analysis demonstrated that the “NaY method” is the most suitable method for the production of measurable quantities of unsymmetrical *meso*-aryl porphyrins, showing a quite favorable cost-to-efficiency ratio. We strongly believe that this type of developments in porphyrin chemistry is the necessary boost for a wider use of unsymmetrical *meso*-aryl substituted porphyrins.

## Figures and Tables

**Table 1:** Comparative study for the synthesis of 5-(4-hydroxyphenyl)-10,15,20-tris(phenyl) porphyrin (**1**) using Adler-Longo, Gonsalves-Pereira, Lindsey, and “NaY” methods.

Method	Reagents (exc. aldehydes and pyrrole)	Temperature	Concentration (M)	Yield (%)
Adler-Longo	propionic acid	140 °C	0.30	6
Gonsalves-Pereira	acetic acid/nitrobenzene	130 °C	0.40	7
Lindsey	dichlorome thanetrifluoroacetic acid DDQ/triethylamine	25 °C, then 45 °C	0.03	15
This work	acetic acid/nitrobenzene NaY zeolite	130 °C	0.42	16

**Table 2:** Calculated E Factors and price for preparation of Porphyrin **1** ^a^.

Method	E Factor	Price (10 mmol)	Price (10 mmol) ^b^	Price (g)
Adler-Longo	6793	1068 €	652 €	103 €
Gonsalves-Pereira	4233	1011 €	741 €	118 €
Lindsey	7090	2002 €	995 €	158 €
NaY	2350	750 €	504 €	80 €

^a^ Prices do not reflect reagents/solvents acquisitions on a large scale, but at a laboratory scale, using a well-established chemical company website prices. ^b^ Considering solvent reutilization (non-mixed reaction and chromatography solvents)

## Supplementary Materials

The following are available online. Supplementary Materials: Characterization of NaY acidity, synthetic procedures for all compounds, characterization data, sustainability factors, and price evaluations.
