# Supplementary material for: A Cost-Efficient Method for Unsymmetrical Meso-Aryl Porphyrin Synthesis Using NaY Zeolite as an Inorganic Acid Catalyst
Source: Molecules. 2017 May 5;22(5):741. doi: 10.3390/molecules22050741 (PMC6154588; doi:10.3390/molecules22050741)
Supplement: Supplementary file 1 [file molecules-22-00741-s001.pdf]

Supplementary Materials: Highly Cost-Efficient Method for Unsymmetrical *meso*-Aryl Porphyrins Synthesis Using NaY Zeolite as Inorganic Acid Catalyst

Mário J. F. Calvete*, Lucas D. Dias, César A. Henriques, Sara M. A. Pinto, Rui M. B. Carrilho and Mariette M. Pereira*

S1. Materials and methods

Commercially available reagents were purchased from Aldrich, Fluorochem and Acros, being used as received. Zeolite NaY was purchased from Zeolyst. All solvents were pre-dried according to standard laboratory techniques. All spectroscopic data from known porphyrins was confirmed (1H-NMR and UV-Vis) and are in agreement with the literature. UV-visible absorption spectra were recorded on a Hitachi U-2010 using quartz cells. The molar absorption coefficients were determined using toluene as solvent. Zeolite acidity measurements were performed using pyridine as probe molecule, followed by Infrared spectroscopy (FTIR); a Nicolet Nexus spectrometer was used for the purpose. 1H NMR spectra were recorded on a 400 MHz Brucker-Amx. The chemical shifts are given in parts per million (ppm) relative to tetramethylsilane at δ 0.00 ppm for proton spectra. Mass spectra were acquired using an Applied Biosystems Voyager DE-STR instrument equipped with a nitrogen laser (λ = 337 nm) or Bruker microTOFQ instrument by Unidade de Masas e Proteomica – Universidade de Santiago de Compostela, Spain. Column chromatographies were performed with silica gel grade 60, 70-230 mesh as stationary phase.

S2. Characterization of acidic properties of NaY zeolite

Acidity measurements were performed using pyridine as probe molecule, followed by Infrared spectroscopy (FTIR); a Nicolet Nexus spectrometer was used for the purpose. In a typical experiment, samples were pressed into thin wafers (10-20 mg/cm^2) and heated in an IR glass cell from room temperature up to 450 ºC (5 ºC.min^{-1}) for 3 h under vacuum (10^{-5} mbar). Afterwards, the samples were cooled down to 150 °C and left in contact with pyridine for 10 min. Then, excess probe molecule was removed for 30 min under vacuum and the IR spectra were recorded; 64 scans with a resolution of 4 cm^{-1} were collected for each spectrum. The concentrations of Brönsted and Lewis sites able to retain the pyridine at 150 ºC were determined using the integrated areas

of the bands at 1541 cm^{-1} and 1445 cm^{-1} [1],[1] respectively, and the extinction coefficients determined by Emeis (Fig. 1) [2].[2]

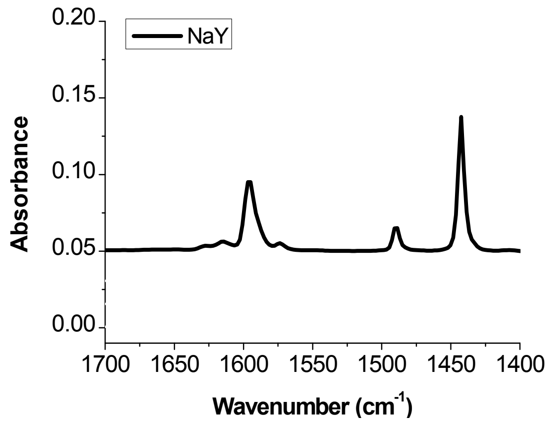

Figure S1. FTIR spectra of NaY obtained after pyridine contact and subsequent desorption under vacuum at 150 ºC for 30 min.

S3. Preparation of 5-(4-hydroxyphenyl)-10,15,20-tris(phenyl) porphyrin (1)

S3.1. NaY method

An amount of 1.0 g of NaY zeolite (0.08 mmol) was introduced into a 50 mL round flask, containing a mixture of 4-hydroxybenzaldehyde (0.625 mmol, 76.3 mg), benzaldehyde (1.875 mmol, 0.19 mL) in a glacial acetic acid/nitrobenzene mixture (7 mL/5 mL). Addition of equimolar amount of pyrrole (2.5 mmol, 0.17 mL) was carried out dropwise under stirring and heating (≈120 °C). After complete addition (ca. 3 min), the suspension was heated further till reflux temperature (≈130 °C) and maintained at this temperature for *ca.* 2 hours. The hot suspension was filtered and the resulting solid material washed with tetrahydrofuran (THF) until no coloured material was collected on the supernatant (250 mL). As alternative Soxhlet extraction with THF can be performed, but in our case we found washing sufficient. The volume of solution was then reduced by rotoevaporation (enough volume to remove the added washing solvent). To induce precipitation, *n*-hexane (*ca.* 50 mL) was added. The Erlenmeyer flask containing the statistical porphyrin mixture was left overnight in the refrigerator and the deposited solid was collected by filtration and then purified by column chromatography using silica gel as stationary phase, starting with *n*-hexane:CH_2Cl_2 (1:3) to collect the first fraction (5,10,15,20-tetraphenyl porphyrin) and then with CH_2Cl_2 to collect the second band. As this fraction was identified as the porphyrin **1**, other following bands were discarded. Fraction 2 was evaporated to dryness and the resulting solid was dried under vacuum and weighed to give 60 mg (0.0938 mmol) of **1** (16% yield). Characterization data is in accordance with the literature [3].[3]

S3.2. Adler-Longo method [4][4]

A mixture of 4-hydroxybenzaldehyde (3.75 mmol, 0.458 g) and benzaldehyde (11.25 mmol, 1.13 mL) was introduced into a 250 mL round flask containing propionic acid (100 mL). Addition of equimolar amount of pyrrole (15 mmol, 1.03 mL) was carried out dropwise under stirring and heating (≈130 °C). After complete addition (ca. 10-12 min), the suspension was heated further till reflux temperature (≈150 °C) and maintained at this temperature for *ca.* 2 hours. The flask condenser was substituted by a distillation apparatus and the solvent mixture was removed by vacuum, under heating. The obtained solid was redissolved in CH_2Cl_2 (30 mL) and the solution washed with saturated $NaHCO_3$ solution (3 x 25 mL). The solution was concentrated to dryness in a rotoevaporator and purified by column chromatography using silica gel as stationary phase, starting with *n*-hexane:CH_2Cl_2 (1:3) to collect the first fraction (5,10,15,20-tetraphenyl porphyrin) and finally with CH_2Cl_2 to collect the second band, contaminated with the corresponding chlorin (close R_f). Other following bands were discarded. Fraction 2 was evaporated to dryness and the resulting solid was dried under vacuum and weighed to give 142 mg (0.225 mmol) of **1** plus its corresponding chlorin (~10%), in an approximated 6% yield.

S3.3. Gonsalves-Pereira method [5] [5]

A mixture of 4-hydroxybenzaldehyde (3.75 mmol, 0.458 g) and benzaldehyde (11.25 mmol, 1.13 mL) was introduced into a 100 mL round flask containing a glacial acetic acid/nitrobenzene mixture (50 mL/25 mL). Addition of equimolar amount of pyrrole (15 mmol, 1.03 mL) was carried out dropwise under stirring and heating (≈120 °C). After complete addition (ca. 10-12 min), the suspension was heated further till reflux temperature (≈130 °C) and maintained at this temperature for *ca.* 1 hour. The flask condenser was substituted by a distillation apparatus and the solvent mixture was removed by vacuum, under heating. The deposited solid was purified by column chromatography using silica gel as stationary phase, starting with *n*-hexane to remove nitrobenzene traces, then *n*-hexane:CH_2Cl_2 (1:3) to collect the first fraction (5,10,15,20-tetraphenyl porphyrin) and finally with CH_2Cl_2 to collect the second band. As this fraction was identified as the porphyrin **1**, other following bands were discarded. Fraction 2 was evaporated to dryness and the resulting solid was dried under vacuum and weighed to give 166 mg (0.263 mmol) of **1** (7% yield).

S3.4. Lindsey method [6] [6]

A mixture of pyrrole (0.25 mL, 3.75 mmol), 4-hydroxybenzaldehyde (114 mg, 0.938 mmol) and benzaldehyde (0.275 mL, 2.75 mmol) in $CHCl_3$ (250 ml) was bubbled with N_2 for 30 min, and TFA

(0.13 mL, 2.0 mmol) was added. The mixture was stirred at room temperature under N_2 for 1 hr. DDQ (625 mg, 3.75 mmol) was added and the mixture was stirred for 12 hr with protection from light. Triethylamine (2.5 ml, 19 mmol) was added and stirred for 30 min, and the solvent was removed by rotary evaporation. The residue was then suspended in CH_2Cl_2, placed on the top of a dry column of Al_2O_3, and eluted with CH_2Cl_2. The eluted solution was evaporated and then purified by chromatography on silica gel, starting with *n*-hexane:CH_2Cl_2 (1:4) to collect the first fraction (5,10,15,20-tetraphenyl porphyrin) and finally with CH_2Cl_2 to collect the second band. As this fraction was identified as the porphyrin **1**, other following bands were discarded. Fraction 2 was evaporated to dryness and the resulting solid was dried under vacuum and weighed to give 89 mg (0.14 mmol) of **1** (15% yield).

S4. Porphyrin yield as function of the amount of zeolite NaY used

Ratio was calculated as the quotient between the number of moles of NaY used per sum of moles of pyrrole and aldehydes (reagents). For instance, when the reaction (see above, point **2**) was performed using 1 g NaY (0.08 mmol) per 5 mmol reagents, the ratio was 0.032 *i.e.*, NaY was present in 3.2% of all reagents. When ratio was 0.032, the yield obtained was *ca.* 16 %. The yields obtained using different ratios of NaY per amount of reagents is presented in Table S1 and Figure S2.

Table S1. Isolated yields of 5-(4-hydroxyphenyl)-10,15,20-tris(phenyl) porphyrin (**1**) vs amount of NaY used.

NaY ratio	Isolated yield (%)
0	7
0.013	9
0.021	11
0.032	16
0.043	16

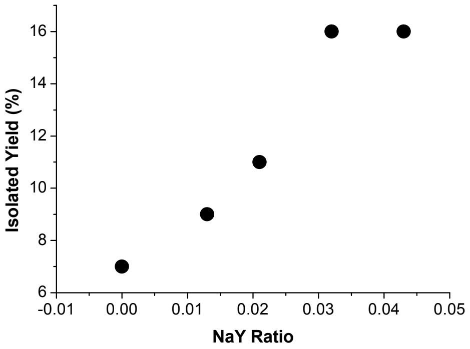

Figure S2 Isolated yields of porphyrin **1** *vs* NaY amount. Note: when amount NaY = 0, it represents the isolated yields using the nitrobenzene method.

S5. Catalyst recycling

The catalyst zeolite NaY was tested in successive cycles to evaluate the reutilisation process in the synthesis of 5-(4-hydroxyphenyl)-10,15,20-tris(phenyl) porphyrin **1**. Using the best determined conditions, after the first cycle, the selected catalyst was collected by filtration and washed with chloroform and tetrahydrofuran, following drying of the solid overnight, at 150 ºC under vacuum. The second, third, fourth and fifth and sixth cycles were carried out with the recovered solid and reactivation procedure was followed for each reutilisation.

Table S2 Reutilisation of NaY on the synthesis of porphyrin **1**

Number of cycles in NaY reutilisation	Isolated yields of porphyrin **1** (%)
1	16
2	15
3	16
4	16
5	15
6	12

S6. Calculation of sustainability E Factors

Density of liquid chemicals used for the calculation of residues (d = density)

Propionic acid d= 990 g/L

Nitrobenzene d= 1200 g/L

Acetic acid d= 1050 g/L

Dichloromethane d= 1330 g/L

Chloroform d= 1489 g/L

Trifluoroacetic acid d= 1489 g/L

n-Hexane d= 655 g/L

Water d= 1000g/L

Triethylamine d= 725.5 g/L

Saturated sodium bicarbonate solution d= 2200 g/L

S6.1. Calculation of E Factor for the preparation of 5-(4-hydroxyphenyl)-10,15,20-tris(phenyl) porphyrin 1, considering the preparation of 10 mmol of end product

Adler-Longo method

E = (4356 g propionic acid + 1771.6 g dichloromethane + 7392.6 g sodium bicarbonate + 2000 g silica + 9310 g dichloromethane + 1965 g n-hexane)/6.3 g porphyrin

E = 6793

Gonsalves-Pereira method

E = (1995 g nitrobenzene + 1140 g acetic acid + 2000 g silica + 9310 g dichloromethane + 1965 g n-hexane)/6.3 g porphyrin

E = 4233

Lindsey method (J. Org. Chem. 1987, 52, 827-836)

E = (29184.4 g chloroform + 15.2 g TFA + 142.2 g triethylamine + 49 g DDQ + 2000 g Al_2O_3 + 2000 g silica + 9310 g dichloromethane + 1965 g n-hexane)/6.3 g porphyrin

E = 7090

Lindsey method (J. Org. Chem. 1994, 59, 579–587)*

E = (3720 g dichloromethane + 160 g BF3Et2O + 160 g TCQ + 2000 g silica + 9310 g dichloromethane + 1965 g n-hexane)/6.3 g porphyrin

E = 2750

*this method was not reproduced but calculations assuming similar yields obtained and similar purification procedures as for the other methodologies were performed.

*NaY method***

784 g nitrobenzene + 641 g acetic acid + 2000 g silica + 9410 g dichloromethane + 1970 g n-hexane)/ 6.3 g porphyrin

E = 2350

**Since NaY is reusable, it is not included in the calculations

S7. Price evaluation related to the synthesis of porphyrin 1

Notes: Market prices were calculated on basis of laboratory scale acquisition at Sigma-Aldrich company and available at www.sigmaaldrich.com, excluding NaY, whose prices were calculated from Zeolyst International company. Prices do not reflect reagents and solvents acquisitions on a large scale, but at a laboratory scale, just following Sigma-Aldrich website prices. Amounts of solvents and reagents used for the preparation of 10 mmol product were calculated simply by multiplying the amounts used in preparations according to the experimental above, considering obtained yields.

Table S3 Price calculation for the preparation of 10 mmol 5-(4-hydroxyphenyl)-10,15,20-tris(phenyl) porphyrin **1** using Adler-Longo method.

Solvents/Reagents	CAS Number	Market Prices[a] (€)	Amount used (10 mmol product)	Price (10 mmol product)
Propionic acid	79-09-4	1L = 69 €	4.44 L	306.36 €
4-hydroxybenzaldehyde	123-08-0	50g = 34.7 €	20.34 g	14.12 €
benzaldehyde	100-52-7	100mL = 60 €	50.17 mL	30.06 €
Pyrrole	109-97-7	100mL = 64.7 €	45.73 mL	29.59 €
$NaHCO_3$	144-55-8	500g = 39.1 €	130 g	10.17 €
Silica gel	112926-00-8	1Kg = 143 €	2 Kg	286 €
n-hexane	110-54-3	2.5L = 84 €	3 L	100.8 €
dichloromethane	75-09-2	2.5L = 104 €	7 L	291.2 €
			**Total**	**1068.3 €**

Table S4 Price calculation for the preparation of 10 mmol 5-(4-hydroxyphenyl)-10,15,20-tris(phenyl) porphyrin **1** using Gonsalves-Pereira method.

Solvents/Reagents	CAS	Market Prices (€)	Amount used (10 mmol product)	Price (10 mmol product)
acetic acid	64-19-7	1 L = 69 €	1.9 L	131.1 €
nitrobenzene	98-95-3	0.5 L = 73.0 €	0.95 L	138.7 €
4-hydroxybenzaldehyde	123-08-0	50 g = 34.7 €	17.40 g	12.08 €
benzaldehyde	100-52-7	100 mL = 60 €	42.94 mL	25.76 €
Pyrrole	109-97-7	100 mL = 64.7 €	39.14 mL	25.32 €
Silica gel	112926-00-8	1 Kg = 143 €	2 Kg	286 €
n-hexane	110-54-3	2.5 L = 84 €	3 L	100.8 €
dichloromethane	75-09-2	2.5 L = 104 €	7 L	291.2 €
			**Total**	**1010.96 €**

Table S5 Price calculation for the preparation of 10 mmol 5-(4-hydroxyphenyl)-10,15,20-tris(phenyl) porphyrin **1** using Lindsey method.

Solvents/Reagents	CAS Number	Market Prices (€)	Amount used (10 mmol product)	Price (10 mmol product)
chloroform	67-66-3	2.5 L = 104 €	19.6 L	815.36 €
4-hydroxybenzaldehyde	123-08-0	50 g = 34.7 €	8.94 g	6.2 €
benzaldehyde	100-52-7	100 mL = 60 €	21.56 mL	12.94 €
Pyrrole	109-97-7	100 mL = 64.7 €	19.6 mL	12.68€
Trifluoracetic acid	76-05-1	100 mL = 58.1 €	10.19 mL	5.92 €
2,3-Dichloro-5,6-dicyano-p-benzoquinone	84-58-2	10 g = 30.2 €	49 g	147.98 €
triethylamine	121-44-8	1 L = 52 €	196 mL	10.19 €
Al_2O_3	1344-28-1	1 Kg = 94 €	2 Kg	188.0 €
Silica gel	112926-00-8	1 Kg = 143 €	2 Kg	286.0 €
n-hexane	110-54-3	2.5 L = 84 €	3 L	100.8 €
dichloromethane	75-09-2	2.5 L = 104 €	10 L	416.0 €
			**Total**	**2002.07 €**

Table S6 Price calculation for the preparation of 10 mmol 5-(4-hydroxyphenyl)-10,15,20-tris(phenyl) porphyrin **1** using "NaY" method.

Solvents/Reagents	CAS	Market Prices (€)	Amount used (10 mmol product)	Price (10 mmol product)
acetic acid	64-19-7	1 L = 69 €	746.7 mL	51.52 €
nitrobenzene	98-95-3	0.5 L = 73.0 €	533.4 mL	38.94 €
4-hydroxybenzaldehyde	123-08-0	50 g = 34.7 €	8.14 g	5.65 €
benzaldehyde	100-52-7	100 mL = 60 €	20.05 mL	12.03 €
Pyrrole	109-97-7	100 mL = 64.7 €	18.13 mL	11.73 €
NaY	1318-02-1	0.5 Kg = 220 €	106.7 g	46.95 €
Silica gel	112926-00-8	1 Kg = 143 €	1.75 Kg	250.2 €
n-hexane	110-54-3	2.5 L = 84 €	2,5 L	84 €
dichloromethane	75-09-2	2.5 L = 104 €	6 L	249.6 €
			**Total**	**750.62 €**

Table S7 Price calculation for the preparation of 10 mmol 5-(4-hydroxyphenyl)-10,15,20-tris(phenyl) porphyrin **1**, disregarding the contribution of reaction solvents (when not mixtures) and chromatography solvents.

Solvents/Reagents	Market Prices (€)	Amount (price) used per reaction (Adler)[a]	Amount (price) used per reaction (Pereira)[a]	Amount (price) used per reaction (Lindsey)[a]	Amount (price) used per reaction (NaY)[a]
acetic acid[b]	1 L = 69 €	--------	250 mL (17.25 €)	--------	175 mL (12 €)
nitrobenzene[b]	0.5 L=73 €	--------	125 mL (18.25€)	--------	125 mL (18.25€)
Propionic acid [a]	1L=69 €	0.4 L (27.6€)	--------	--------	--------
Chloroform [a]	2.5 L = 104 €	--------	--------	1.25 L (52€)	--------
NaY [a]	0.5 Kg=220 €	--------	--------	--------	25 g (11€)
4-hydroxy-benzaldehyde[b]	50g=34.7 €	2.284 g (1.6€)	2.284 g (1.6€)	0.57 g (0.4€)	1.9 g (1.3€)
benzaldehyde[b]	100mL=60 €	5.65 mL (3.1€)	5.65 mL (3.1€)	1.375 mL (0.8€)	4.7 mL (2.8€)
pyrrole[b]	100mL=65 €	5.15 mL (3.3€)	5.15 mL (3.3€)	1.25 mL (0.8€)	4.25 mL (2.8€)
TFA[b]	100 mL=58 €	--------	--------	0.65 mL (0.4€)	--------
DDQ[b]	10 g = 30 €	--------	--------	3.125 g (10€)	--------
Triethylamine[b]	1 L = 52 €	--------	--------	12.5 mL (0.65€)	--------
$NaHCO_3$[b]	500g=39.1 €	15 g (1.2€)	--------	--------	--------
Al_2O_3, [b]	1 Kg = 94 €	--------	--------	0.15 Kg	--------

				(14.1€)	
Silica gel[b]	1Kg=143 €	0.3 Kg (42.8€)	0.3 Kg (42.8€)	0.2 Kg (28.6€)	0.25 Kg (36€)
n-hexane [a]	2.5L = 84 €	0.5 L (16.8€)	0.5 L (16.8€)	0.4 L (13.4€)	0.4 L (13.4€)
dichloromethane [a]	2.5L = 104 €	3.5 L (145.6€)	3.5 L (145.6€)	3.2 L (133.1€)	3.2 L (133.1€)
	Yield	1.125 mmol	1.315 mmol	0.7 mmol	2.35 mmol
	Factor for 10 mmol	x 8.89	x 7.6	x 14.3	x 4.26
	total	**652 €**	**741 €**	**995 €**	**504 €**

[a] items which do not need multiplication by Factor, since they are recoverable

[b] not recoverable items

Table S8 E Factors and prices calculated for the synthesis of 5-(4-hydroxyphenyl)-10,15,20-tris(phenyl) porphyrin **1** using several methods.

Method	E Factor	Price (10 mmol product)	Price (10 mmol product) considering solvent reutilization
Adler-Longo	6793	1068.30 €	652 €
Gonsalves-Pereira	4233	1010.96 €	741 €
Lindsey	7090	2002.07 €	995 €
NaY	2350	750.62 €	504 €

S9. Synthesis of unsymmetrical *meso*-substituted porphyrins 2 – 6

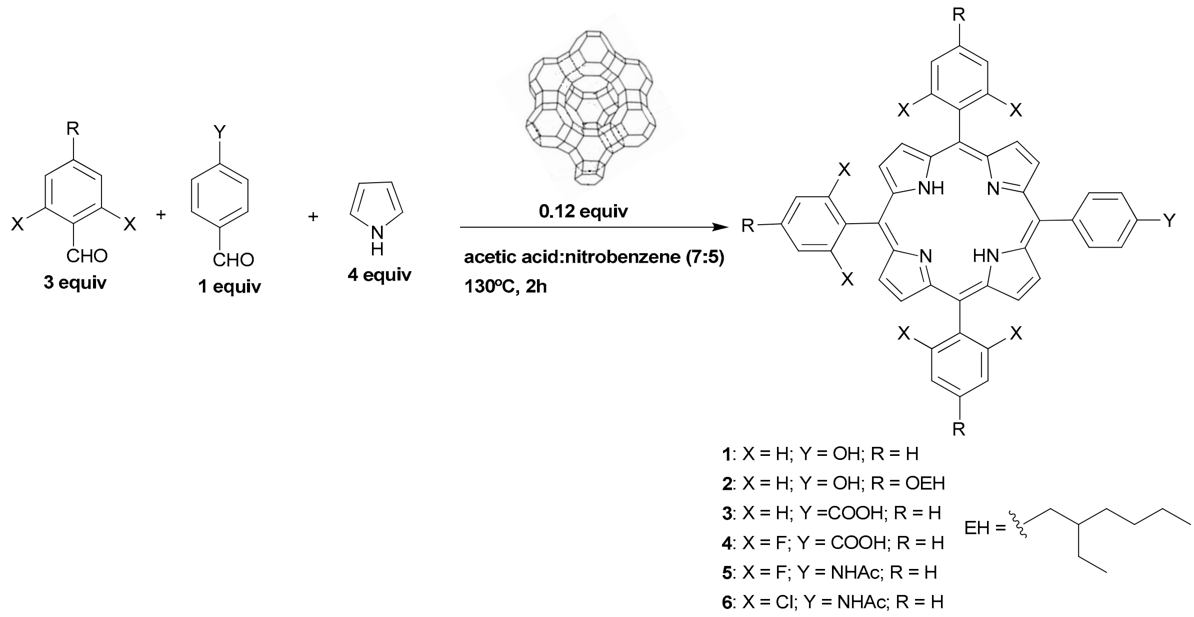

Scheme 1 Synthesis of unsymmetrical *meso*-aryl substituted porphyrins.

S9.1. General procedure

An amount of 1.0 g of NaY zeolite (0.08 mmol) was introduced into a 50 mL round flask, containing a 1:3 mixture of aldehydes (0.625 mmol:1.875 mmol) in a glacial acetic acid/nitrobenzene mixture (7 mL/5 mL). Addition of equimolar amount of pyrrole (2.5 mmol, 0.17 mL) was carried out dropwise under stirring and heating (≈120 °C). After complete addition (ca. 3 min), the suspension was heated further till reflux temperature (≈130 °C) and maintained at this temperature for *ca.* 2 hours. The hot suspension was filtered and the resulting solid material washed with tetrahydrofuran (THF) until no coloured material was collected on the supernatant (250 mL). As alternative Soxhlet extraction with THF can be performed, but in our case we found washing sufficient. The volume of solution was then reduced by rotoevaporation (enough volume to remove the added washing solvent). To induce precipitation, *n*-hexane (*ca.* 50 mL) was added. The Erlenmeyer flask containing the statistical porphyrin mixture was left overnight in the refrigerator and the deposited solid was collected by filtration and then purified by column chromatography using silica gel as stationary phase, starting with *n*-hexane:CH_2Cl_2 (1:3) and then increasing polarity using appropriate gradients of *n*-hexane:CH_2Cl_2, then pure CH_2Cl_2 and finally CH_2Cl_2:ethanol gradients if necessary to collect fraction 2 (the target compound). This fraction was then evaporated to dryness and the resulting solid was dried under vacuum and weighed.

S9.2. 5-(4-hydroxyphenyl)-10,15,20-tris(4-ethylhexyloxyphenyl) porphyrin (2)

Benzaldehydes used: 4-hydroxybenzaldehyde (0.625 mmol, 76.3 mg) and 4-ethylhexyloxybenzaldehyde[7] (1.875 mmol, 439 mg). Column chromatography was carried out using as eluent *n*-hexane:CH_2Cl_2 (1:3) to collect the first fraction (A_4 product) followed by CH_2Cl_2 to collect porphyrin **2**. We obtained 95 mg of porphyrin **2** (15% yield). Characterisation data is in agreement with the literature.[7,8]

S9.3. 5-(4-carboxyphenyl)-10,15,20-tris(phenyl) porphyrin (3)

Benzaldehydes used: 4-carboxybenzaldehyde (0.625 mmol, 94 mg) and benzaldehyde (1.875 mmol, 200 mg). Column chromatography was carried out using as eluent *n*-hexane:CH_2Cl_2 (1:3) to collect the first fraction (A_4 product) followed by ethanol:CH_2Cl_2 (1:200) to collect porphyrin **3**. We obtained 70 mg of porphyrin **3** (17% yield). Characterisation data is in agreement with the literature.[3]

S9.4. 5-(4-carboxyphenyl)-10,15,20-tris(2,6-difluorophenyl) porphyrin (4)

Benzaldehydes used: 4-carboxybenzaldehyde (0.625 mmol, 94 mg) and 2,6-difluorobenzaldehyde (1.875 mmol, 267 mg). Column chromatography was carried out using as eluent *n*-hexane:CH_2Cl_2 (1:3) to collect the first fraction (A_4 product) followed by ethanol:CH_2Cl_2 (1:100) to collect porphyrin **4**. We obtained 62 mg of porphyrin **4** (13% yield). Characterisation data is in agreement with the literature.[9]

S9.5. 5-(4-acetylaminophenyl)-10,15,20-tris(2,6-difluorophenyl) porphyrin (5)

Benzaldehydes used: 4-acetylaminobenzaldehyde (0.625 mmol, 102 mg) and 2,6-difluorobenzaldehyde (1.875 mmol, 267 mg). Column chromatography was carried out using as eluent *n*-hexane:CH_2Cl_2 (1:3) to collect the first fraction (A_4 product) followed by ethanol:CH_2Cl_2 (1:100) to collect porphyrin **5**. We obtained 78 mg of porphyrin **5** (16% yield).

^{1}H NMR ($CDCl_3$, 400 MHz), δH, = ppm 8.90-8.83 (m, 8H, β-H), 8.17 (d, *J* = 8.0 Hz, 2H, Ph(Ac)-H), 7.90 (d, *J* = 8.0 Hz, 2H, Ph(Ac)-H), 7.89-7.76 (m, 3H, Ph-H), 7.49-7.36 (m, 6H, Ph-H), 2.36 (s, 3H, CH_3), -2.75 (s, 2H, NH). **^{13}C NMR** (101 MHz, $CDCl_3$) δC = ppm 134.9, 130.8, 129.1, 119.7, 118.0, 111.5, 111.2, 24.6. **^{19}F NMR** (376.5 MHz, $CDCl_3$) δF = ppm -108.2, -108.3.

HRMS (ESI-FIA-TOF): Calcd. for $C_{46}H_{28}F_6N_5O$: 780.2193; Found *m/z*= 780.2202 $[M]^+$

UV-vis (toluene): λ_{max}, nm (ε, $M^{-1}\cdot cm^{-1}$) 420 ($3.5x10^5$), 515 ($2.3x10^4$), 546 ($6.2x10^3$), 591 ($6.9x10^3$), 656 ($4.3x10^3$).

EA: Anal. Calcd for $C_{46}H_{28}F_6N_5O$: C, 70.86; H, 3.49; N, 8.98. Found: C, 70.87; H, 3.49; N, 8.97.

S9.6. 5-(4-acetylaminophenyl)-10,15,20-tris(2,6-dichlorophenyl) porphyrin (6)

Benzaldehydes used: 4-acetylaminobenzaldehyde (0.625 mmol, 102 mg) and 2,6-dichlorobenzaldehyde (1.875 mmol, 328 mg). Column chromatography was carried out using as eluent *n*-hexane:CH_2Cl_2 (1:3) to collect the first fraction (A_4 product) followed by ethanol:CH_2Cl_2 (1:100) to collect porphyrin **6**. We obtained 71 mg of porphyrin **6** (13% yield).

^{1}H NMR (400 MHz, $CDCl_3$), δH = ppm 8.85 (d, *J* = 4.5 Hz, 2H, β-H), 8.66 – 8.62 (m, 6H, β-H), 8.12 (d, *J* = 8.0 Hz, 2H, Ph(Ac)-H), 7.85 (d, *J* = 7.9 Hz, 2H, Ph(Ac)-H), 7.79-7.65 (m, 9H, Ph-H), 2.31 (s, 3H, CH_3), -2.56 (s, 2H, NH). **^{13}C NMR** (101 MHz, $CDCl_3$) δC = ppm 135.1, 131.2, 129.2, 124.6, 119.7, 117.6, 111.6, 111.4, 24.8.

MS (MALDI-TOF): *m/z*= 879.039 $[M]^+$

UV-vis (toluene): λ_{max}, nm (ε, $M^{-1}\cdot cm^{-1}$) 420 ($3.6x10^5$), 514 ($2.3x10^4$), 545 ($6.1x10^3$), 591 ($6.9x10^3$), 656 ($4.4x10^3$).

EA: Anal. Calcd. for $C_{46}H_{27}F_6N_5O$: C, 62.89; H, 3.10; N, 7.97. Found: C, 62.85; H, 3.08; N, 7.99.

S9.7. NMR spectra of compounds 4 and 5.

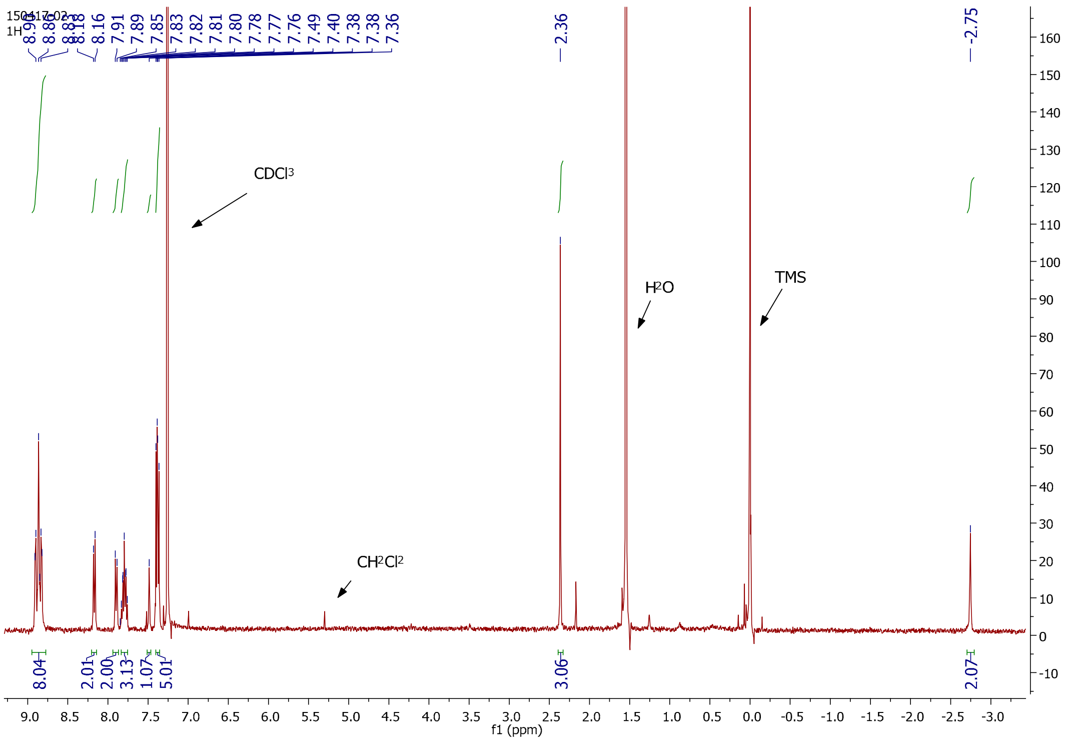

Figure S3 ^{1}H NMR spectrum of compound **5**, recorded in $CDCl_3$.

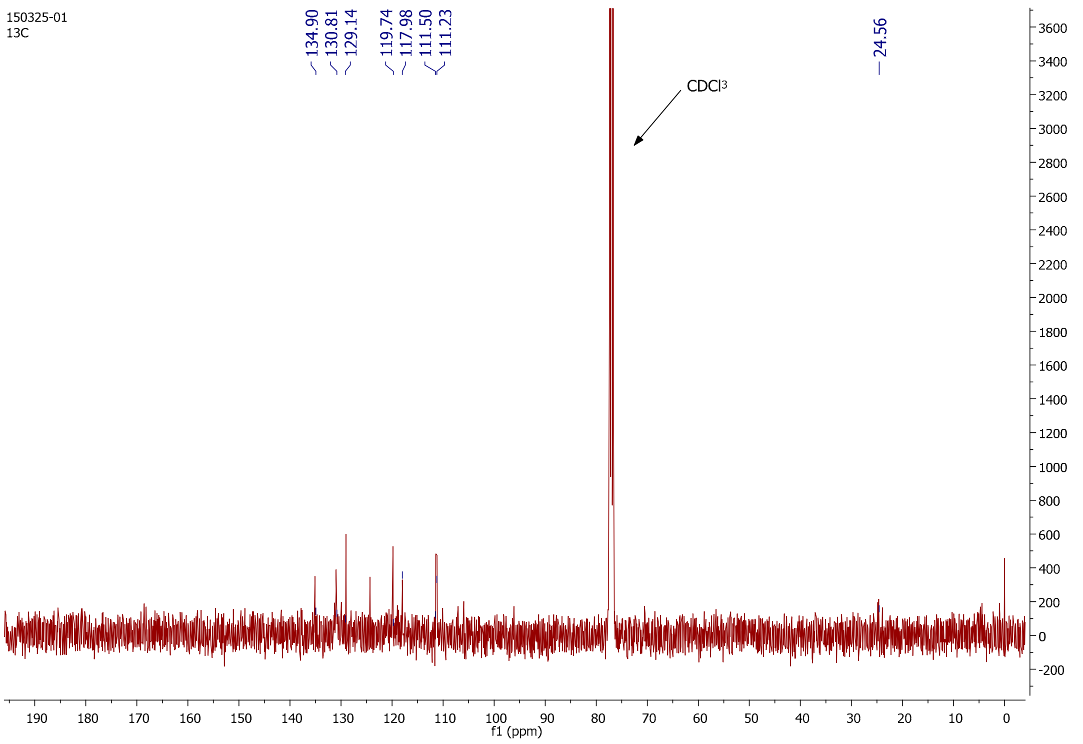

Figure S4 ^{13}C NMR spectrum of compound **5**, recorded in $CDCl_3$.

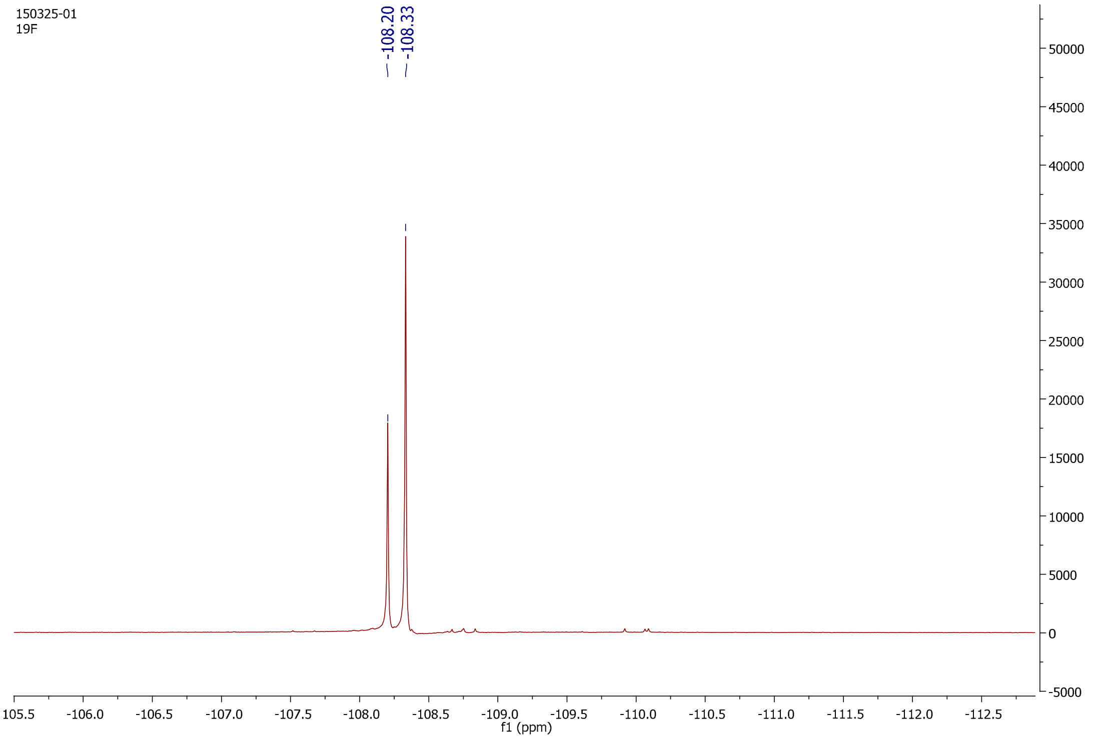

Figure S5 ^{19}F NMR spectrum of compound **5**, recorded in $CDCl_3$.

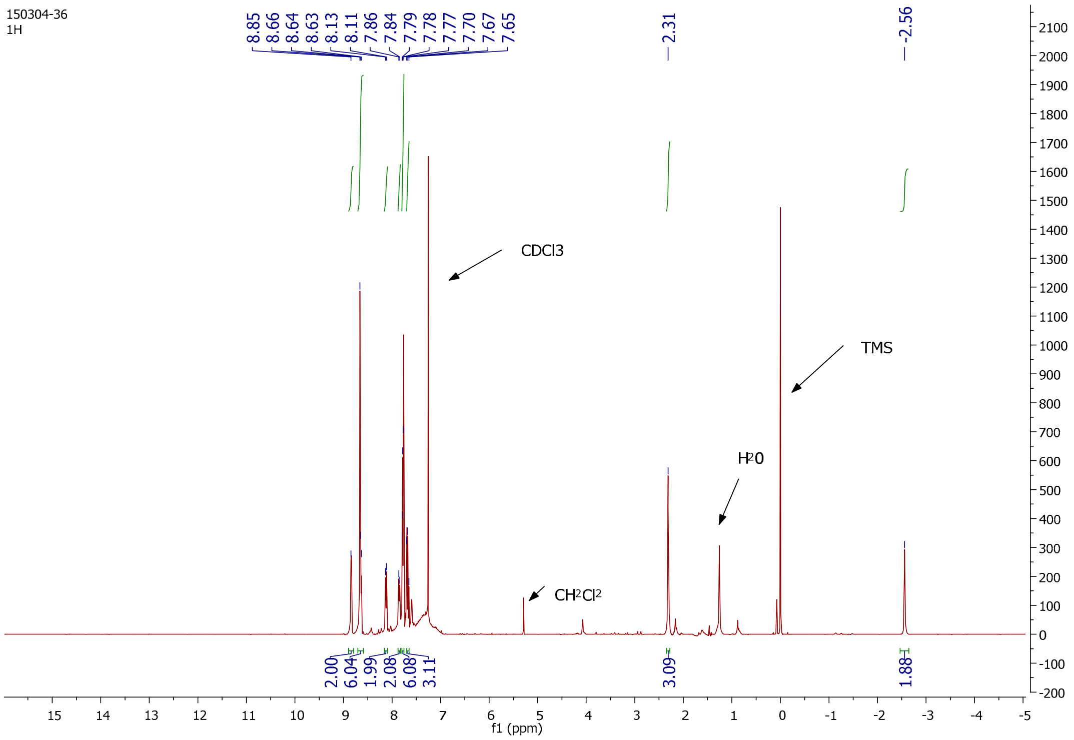

Figure S6 ^{1}H NMR spectrum of compound **6**, recorded in $CDCl_3$.

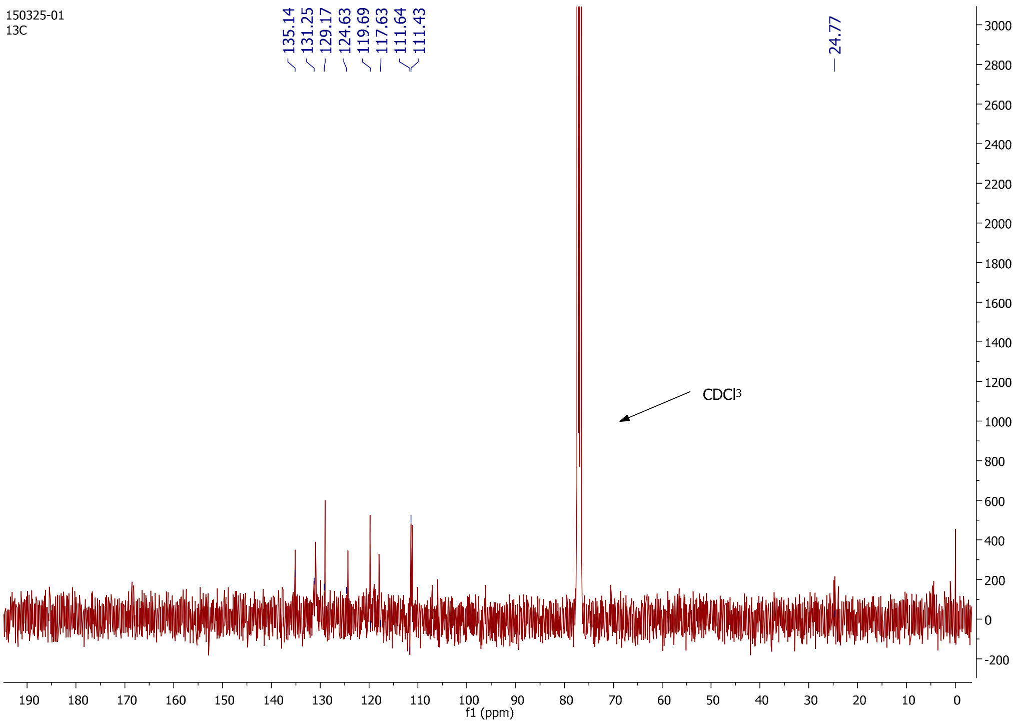

Figure S7 ^{13}C NMR spectrum of compound **6**, recorded in $CDCl_3$.

S10. Mass Spectra of compounds 5 and 6.

RIAIDT
UNIDADE DE ESPECTROMETRIA DE MASAS E PROTEOMICA
Edificio CACTUS * Campus Vida
15782 Santiago de Compostela (Spain)
Tel. 981 563 100. Ext. 16242 * Fax 981 528098
E-mail: sxemasas@usc.es
Web: http://www.ti.usc.es/riaidt/masas/masas.htm

ESPECTRO ESI-FIA-TOF-Medida de Masas Exactas

Analysis Info

Sample Name	SMP100PD_779_1-B,2_01_16575.d	Acquisition Date	5/5/2015 2:02:56 PM
Method	fia_pos_medio_350-1500_egf.m	Instrument	micrOTOF

Medida De Masas Exactas

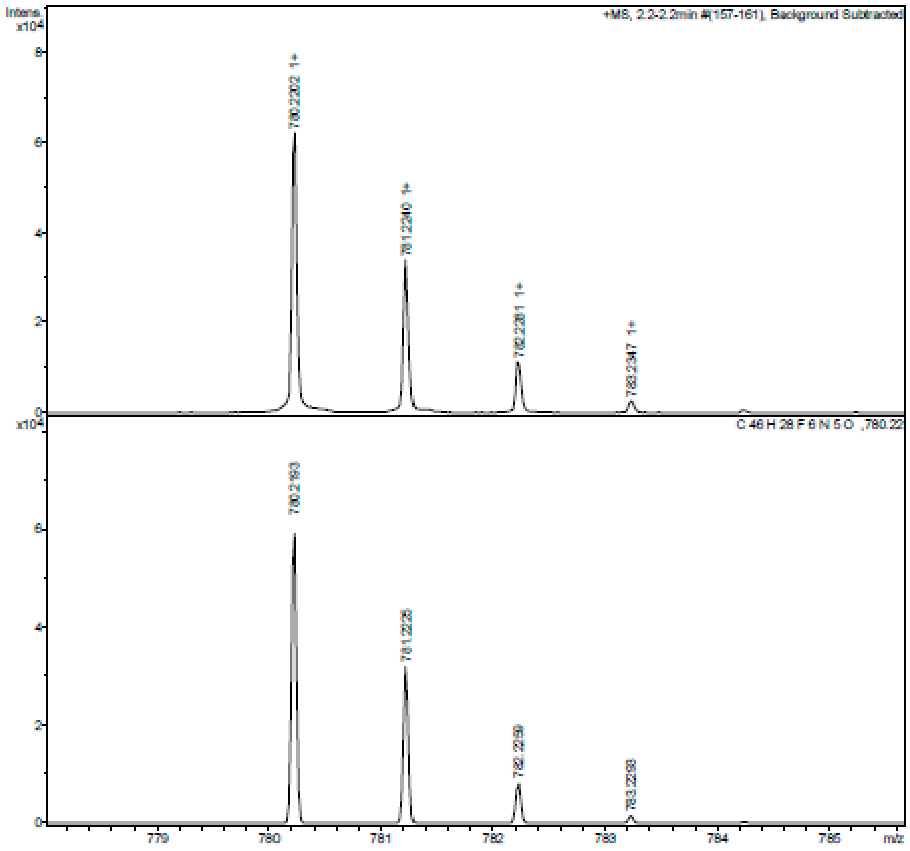

Meas. m/z	#	Formula	Score	m/z	err [mDa]	err [ppm]	mSigma	rdb	e⁻ Conf	N-Rule
780.2202	1	C 46 H 28 F 6 N 5 O	100.00	780.2193	-0.9	-1.2	30.6	32.5	even	ok

Figure S8 Mass spectrum of compound **5**.

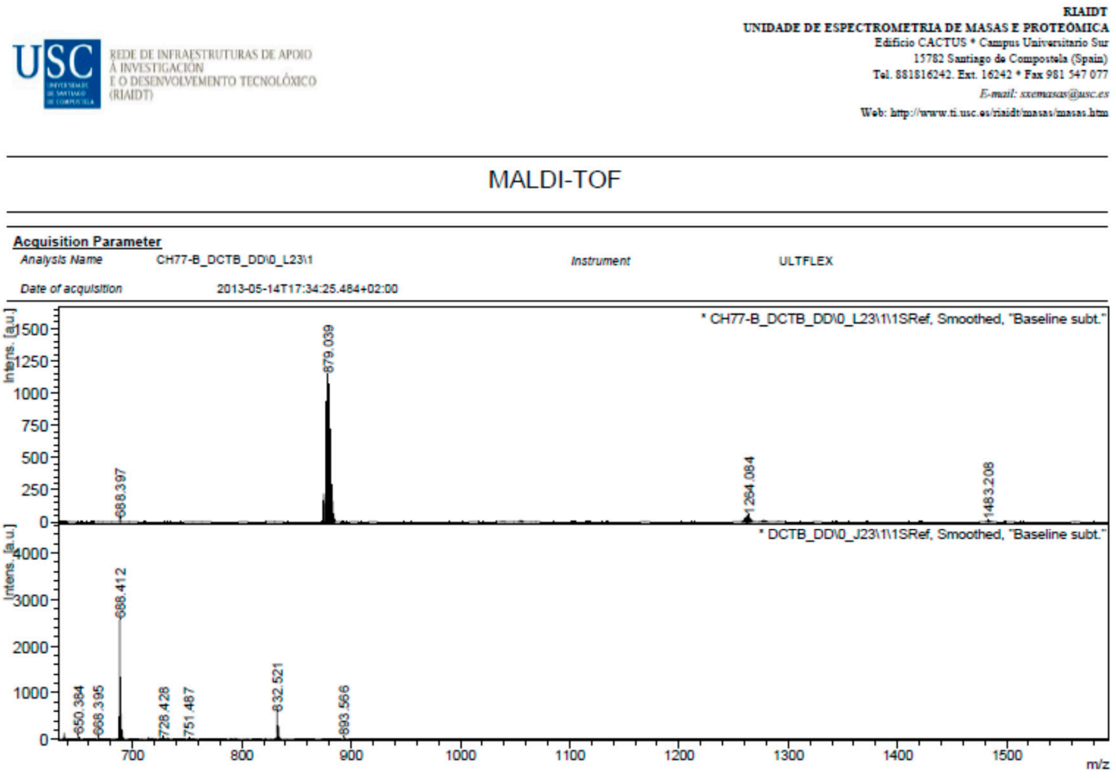

Figure S9 Mass spectrum of compound **6**.

S11. UV-Vis Spectra of compounds 5 and 6.

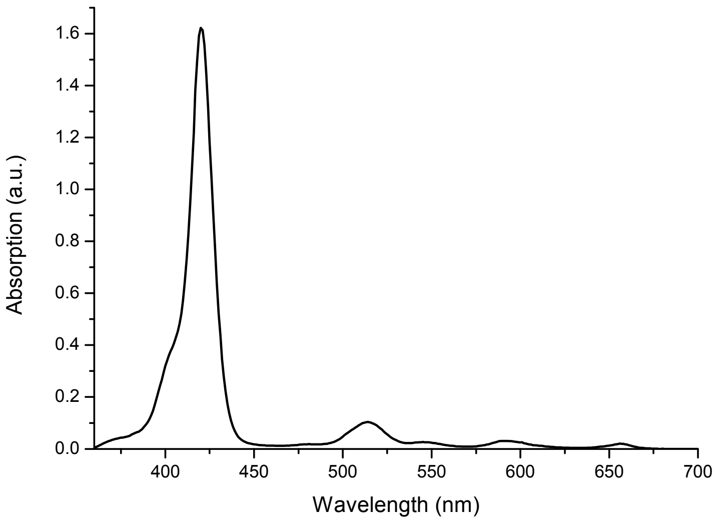

Figure S10. Absorption spectrum of compound **5**, recorded in toluene.

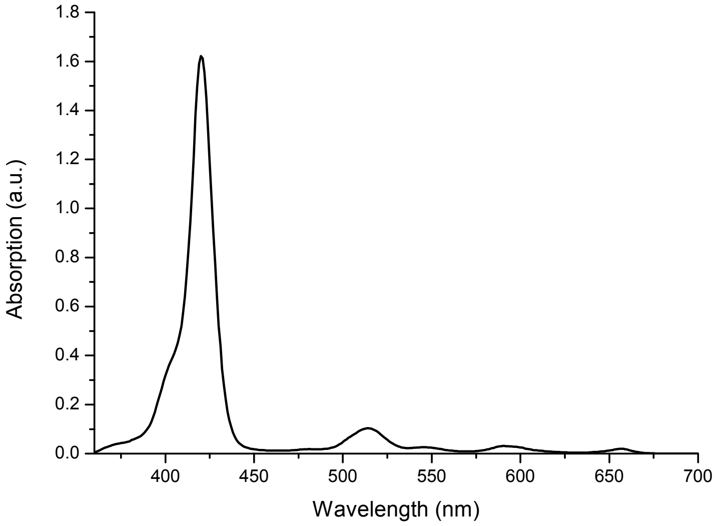

Figure S11. Absorption spectrum of compound **6**, recorded in toluene.

1 F. Maugé, A. Sahibed-Dine, M. Gaillard and M. Ziolek, *J. Catal.*, 2002, **207**, 353.
2 C.A. Emeis, *J. Catal.*, 1993, **141**, 347.
3 J. P. C. Tome, M. G. P. M. S. Neves, A. C. Tome, J. A. S. Cavaleiro, A. F. Mendonça, I. N. Pegado, R. Duarte, M. L. Valdeira, *Bioorg. Med. Chem.*, 2005, **13**, 3878.
4 A. D. Adler, F. R. Longo, J. D. Finarelli, J. Goldmacher, J. Assour and L. Korsakoff, *J. Org. Chem.*, 1967, **32**, 476.
5 A. M. A. R. Gonsalves, J. M. T. B. Varejao and M. M. Pereira, *J. Heterocycl. Chem.*, 1991, **28**, 635.
6 J. S. Lindsey, I. C. Schreiman, H. C. Hsu, P. C. Kearney and A. M. Marguerettaz, *J. Org. Chem.*, 1987, **52**, 827.
7 A. Henriques, S. M. A. Pinto, M. Pineiro, J. Canotilho, M. E. S. Eusébio, M. M. Pereira and M. J. F. Calvete, *Rsc Adv.*, 2015, **5**, 64902.
8 C. A. Henriques, S. M. A. Pinto, G. L. B. Aquino, M. Pineiro, M. J. F. Calvete and M. M. Pereira, *ChemSusChem*, 2014, **7**, 2821.
9 H. Kon and T. Nagata, *Chem. Eur. J.*, 2012, **18**, 1781.
